# Comparison of bleb morphologies between phacoemulsification combined with Ex-PRESS mini shunt implantation, phacotrabeculectomy and trabeculectomy alone: a two-year retrospective in vivo confocal microscopy study

**DOI:** 10.1186/s12886-024-03364-2

**Published:** 2024-03-06

**Authors:** Yuqiao Zhang, Chunxin Lai, Suwen Zhao, Ling Li, Xiaoyang Luo, Yanlei Chen, Yongyi Niu, Yongjie Qin, Hongyang Zhang

**Affiliations:** 1 Department of Ophthalmology, Nanfang Hospital, Southern Medical University, No. 1838 Guangzhou Dadao North Road, 510000 Guangzhou, China; 2 Department of Ophthalmology and Visual Sciences, The Chinese University of Hong Kong, Hong Kong, SAR China; 3 Department of Ophthalmology, Guangdong Eye Institute, Guangdong Provincial People’s Hospital, Guangzhou, China; 4 Shantou University Medical College, Shantou, China; 5 The Second School of Clinical Medicine, Southern Medical University, Guangzhou, China

**Keywords:** Primary open angle glaucoma, Glaucoma drainage implant, Phacoemulsification, Trabeculectomy, In vivo confocal microscopy

## Abstract

**Background:**

To compare the bleb morphologies of phacoemulsification combined with Ex-PRESS implantation (Phaco-ExPRESS), phaco trabeculectomy (Phaco-Trab), and trabeculectomy (Trab) in postoperative two years.

**Methods:**

Patients with primary open-angle glaucoma (POAG) with or without cataracts were included in this study. All patients underwent surgeries of either Phaco-ExPRESS, Phaco-Trab, or Trab. The morphologic structures of the filtering bleb, including microcysts area, hyperreflective dot density, and stromal connective tissue under in vivo confocal microscope (IVCM), were compared between the three groups. The data were collected preoperatively and postoperatively at 2 weeks, 1 month, 3 months, 6 months, 12 months, 18 months, and 24 months.

**Results:**

Eighty-nine eyes from 89 patients were enrolled, including 32 in the Phaco-ExPRESS group, 25 in the Phaco-Trab group, and 32 in the Trab group. In a 24-month follow-up, bleb morphologies in Phaco-ExPRESS were similar to the Trab group. The area of epithelial microcysts was significantly increased in Phaco-ExPRESS and Trab groups while significantly decreased in Phaco-Trab. At postoperative 24 months, the complete success rate was 65.1% in Phaco-ExPRESS, 32.0% in Phaco-Trab, and 59.4% in the Trab group (*P* = 0.03). The phaco-Trab group had more postoperative anti-glaucoma medications than the other two groups (*P* < 0.05).

**Conclusions:**

Phaco-ExPRESS group and Trab group had similar blebs morphologies in IVCM, with larger microcyst area, looser connective tissue, and less inflammation than Phaco-Trab, indicating that the function of blebs in the Phaco-ExPRESS and Trab group, was more potent than that of Phaco-Trab. All these surgical methods provided adequate IOP control, but Phaco-Trab required more anti-glaucoma medications.

## Introduction

Trabeculectomy is the gold standard of filtration surgery that lowers the intraocular pressure (IOP) to prevent the progression of visual field loss [1]. However, the coincidence of cataracts and glaucoma makes it challenging to treat two diseases simultaneously [2]. The trabeculectomy can lead to the progression of cataract [3], while the subsequent cataract extraction can jeopardize the blebs’ function [4, 5]. However, the IOP reduction after cataract surgery is insufficient and may lead to the progression of visual field defect [6, 7]. Thus, it is preferable to perform combined trabeculectomy and phacoemulsification surgery (Phaco-Trab) [8]. Nevertheless, the Phaco-Trab showed less diffuse blebs and more flat blebs when compared with the trabeculectomy alone at 3 or 6 months postoperatively [9, 10]. The possible reason is that additional cataract surgery may result in intensive anterior chamber inflammation, and thus scarring of filtering blebs and compromise of filtrating function [11, 12]. Therefore, it is still controversial regarding the treatment for coincident cataracts and glaucoma.

Over the last two decades, glaucoma drainage devices have been developed for the treatment of glaucoma. As an alternative surgery to traditional trabeculectomy, the implantation of Ex-PRESS glaucoma mini shunt (Alcon, FortWorth, TX, USA) showed comparable effectiveness to the trabeculectomy [13, 14]. Notably, the Ex-PRESS glaucoma mini shunt implantation results in less traumatization of ocular tissue and thus less postoperative inflammation [15, 16]. A retrospective study showed that the blebs height of the Ex-PRESS glaucoma mini shunt implantation were more diffuse at postoperative 3–18 months than the trabeculectomy, based on the Moorfields Bleb Grading System [17]. Previous studies showed that the filtration blebs created by implantation of ExPRESS showed similar bleb vascularity and bleb survival time [18] but thicker bleb wall than blebs created by trabeculectomy [18, 19]. However, comparison of bleb excision or needling rate between trabeculectomy and ExPRESS group remains controversial [17, 20–26]. Our previous study showed that when compared with the phaco trabeculectomy, the Ex-PRESS glaucoma mini shunt implantation created a more diffused and elevated bleb, with higher density of epithelial microcysts, less inflammation, and looser connective tissue [27] than the blebs created by phacotrabeculectomy. However, compared with the classical trabeculectomy, the bleb morphology and efficacy of the phacoemulsification combined with the Ex-PRESS glaucoma mini shunt (Phaco-ExPRESS) remains unknown [28–31]. Therefore, to better investigate the bleb morphology of the Phaco-ExPRESS, we conducted in vivo confocal microscopy and anterior-segment optical coherence tomography (AS-OCT) to compare the structure of filtering blebs from the trabeculectomy, the Phaco-ExPRESS, and the Phaco-Trab.

## Methods

This is a retrospective study, which was carried out in accordance with the tenets of the Declaration of Helsinki. The study recruited consecutive patients who received glaucoma surgery in Guangdong General Hospital between May 2019 and July 2022. Written informed consent was obtained from all participants.

### Eligibility criteria

Patients with primary open-angle glaucoma (POAG) who had undergone the trabeculectomy, the Phaco-Trab and the Phaco-ExPRESS with at least 24 months of follow-up data were included in this study. Exclusion criteria were: (1) Patients who were diagnosed with angle-closure, neovascular or uveitic glaucoma; (2) Patients who had their procedures less than 24 months ago; (3) Patients who had other previous intraocular surgery, systemic or topical therapies other than anti-glaucoma, ocular surface contact lens wearing in 6 months before the glaucoma surgery; (4) Patients with the preoperative active inflammatory status of the anterior ocular segment. Both eyes of eligible patients were included in the study if they all met the inclusion criteria.

### Patients visits

Baseline demographic information including gender, age, best corrected visual acuity (BCVA), IOP (Goldmann applanation tonometry), central corneal thickness (SW-1000P, Suowei, China), visual field mean deviation (MD) and pattern standardized deviation (PSD) measured by standard automated perimetry (Humphrey Field Analyzer II 750; 24 − 2 Swedish interactive threshold algorithm, Carl Zeiss Meditec, Dublin, CA, USA), number of glaucoma medications were collected.

Postoperative data were collected from the enrolled patients at 2 weeks, 1 month, 3 months, 6 months, 12 months, 18 months, and 24 months. In each time point, BCVA, IOP, slit-lamp anterior segment photography, fundus color stereo photography (Canon, Tokyo, Japan), AS-OCT (RTVue-XR Avanti; Optovue, Fremont, CA, USA, version 2016.2.035) and in vivo confocal microscopy (HRT II Rostok Cornea Module; Heidelberg Engineering, Heidelberg, Germany) were collected.

In accordance with recommendations described in the previous study [32], complete success was defined as IOP < 18 mmHg without anti-glaucoma medications, while qualified success was defined as IOP < 18 mmHg with or without anti-glaucoma medications.

### Surgery Procedure

All surgeries were performed under local anesthesia (Proparacaine hydrochloride 0,5%, Alcon) by a single surgeon (HYZ). The surgeon determined the selection of patients to have the combined procedure or the trabeculectomy alone, considering the degree of visual impairment resulting from cataracts. Patients’ preferences determined the selection of two different types of combined surgery after explaining the mechanism of surgery and potential surgical complications to patients.

The trabeculectomy was performed with a conjunctival flap and a rectangular 5 × 4 mm half-thickness scleral flap. Anti-fibrotic mitomycin (MMC) was applied on several sponges into the subconjunctival space after the scleral flap was created before entering the anterior chamber. Doses of 0.4 mg/ml were used for 3 min. Subsequently, the sponges were removed, and the space between the flap and episcleral was copiously irrigated with a balanced salt solution. A blade was used to enter the anterior chamber, and a peripheral iridectomy is created. After that, releasable and adjustable 10 − 0 nylon sutures were used to close the scleral flap. The conjunctiva was then closed with 10 − 0 nylon sutures.

In the cases of the Phaco-Trab and the Phaco-ExPRESS, a twin site procedure of the phacoemulsification of cataract and acrylic intraocular lens (Akeros Advanced Ai, Bausch & Lomb) implantation was performed after the creation of the scleral flap. In Phaco-Trab and Phaco-ExPRESS group, the same doses (0.4 mg/ml) and the same time duration (3 min) of MMC were applied in the subconjunctival space after creation of the scleral flap. In the Phaco-ExPRESS, rather than entrancing the anterior chamber by a blade, a 25-ga. needle was horizontally inserted into the anterior chamber from the sclera-cornea transition zone parallel to the iris. The Ex-PRESS of model P50 (Alcon, Fort Worth, TX) was then inserted into the anterior chamber. The sutures of the scleral and conjunctival flap were the same as trabeculectomy.

All the eyes underwent surgery received topical steroids for 4 weeks (dexamethasone eye drops four times a day, dexamethasone 0.15% eye ointment once daily) and topical antibiotic for the initial two weeks (levofloxacin 5 mg/mL eye drops four times a day).

### Assessment of filtering bleb

**Fig. 1:**
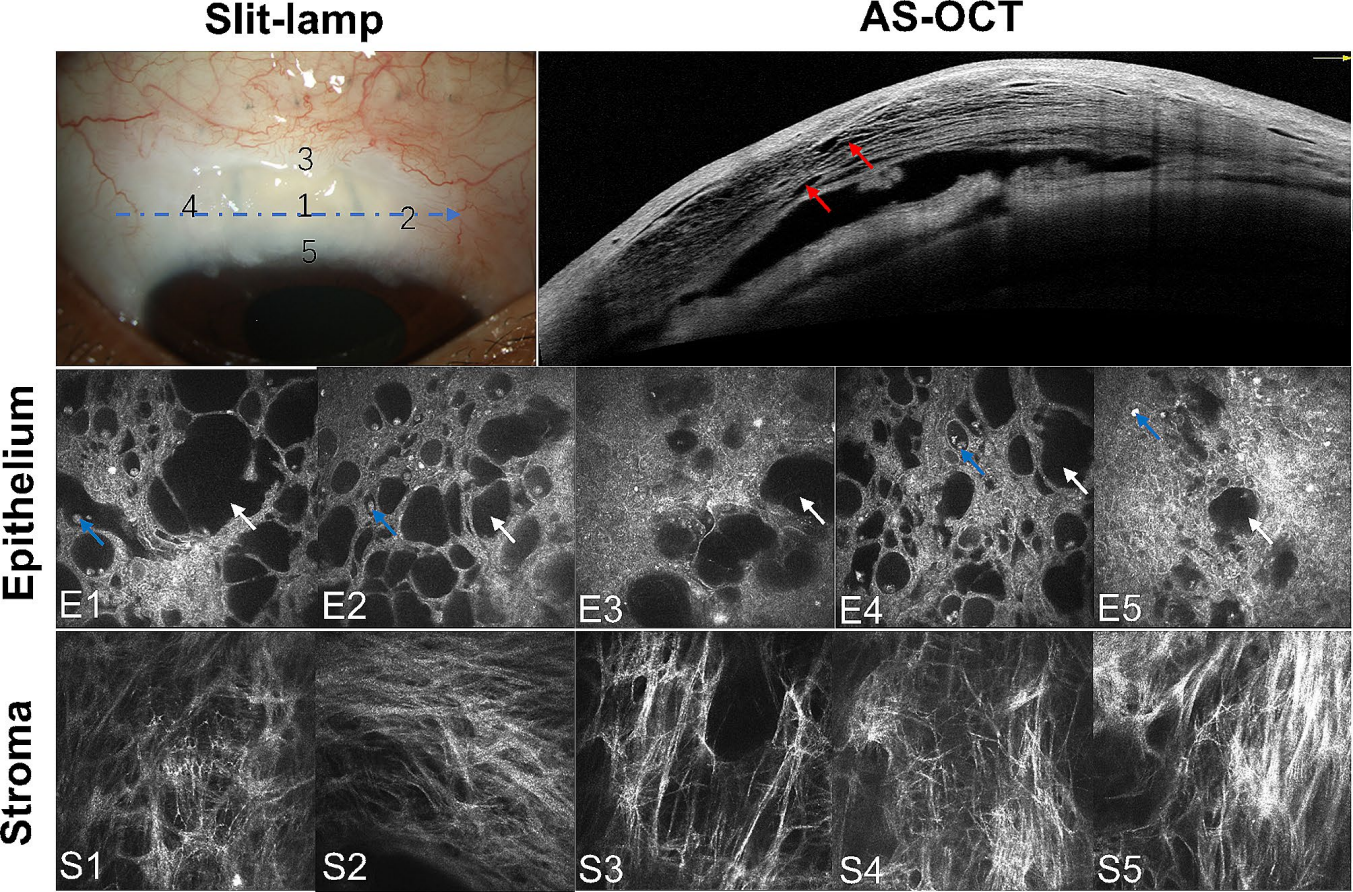
Assessment of filtering blebs. This figure showed the assessment of filtering bleb by slit-lamp, anterior segment optical coherence tomography (AS-OCT) and in vivo confocal microscope (IVCM).The striping phenomenon (red arrows), which showed multiple parallel hyperreflective areas inside the presumed Tenon’s layer, was recorded in each filtering bleb. After taking AS-OCT photograph, scanning involving the 5 sites, numbered 1 to 5 in the slit-lamp photograph, was conducted by IVCM. The AS-OCT scanning was taken across the blue line, as shown in the slit-lamp photograph. For each site, the microcysts area (white arrows) and hyperreflective dots (blue arrows) in the epithelium (E1-E5) and connective tissue in the stroma (S1-S5) were recorded for evaluation

The microstructures of the filtering bleb, including epithelial microcyst, hyperreflective dots, and stromal connective tissue, were documented by in vivo confocal microscope (IVCM, HRT III Heidelberg Engineering, Germany). Five IVCM images were obtained from 5 regions of filtering bleb. Image J software was utilized to calculate the mean epithelial area per µm^2^ of microcyst and density of hyperreflective dot per mm^2^ from 5 regions of filtering bleb. The stromal connective tissue density was calculated by the average gray value of the selected image using Image J software. According to a previous study [33], the average grayscale of stroma, which corresponded to the sum of gray values of all pixels in the entire image divided by the number of pixels, was evaluated for the grading of connective tissue. The grade of connective tissue density was defined as loose (grade 1: gray value < 90.0), mildly dense (grade 2: 90.01 ≤ gray value < 105.00), moderately dense (grade 3: 105.01 ≤ gray value < 125.00), and highly dense (grade 4: gray value ≥ 125.01). A single operator conducted all the IVCM measurements. One operator selected the most represented images from each region, and two other observers (YQZ and CXL) were responsible for image quantification or qualitative measurement. All the analysis methods are summarized in Fig. 1. AS-OCT also assessed all the filtering blebs to investigate the presence of the striping phenomenon, which is characterized by multiple hyperreflective areas inside the presumed Tenon’s layer [34]. The data of AS-OCT was excluded when agreement on the presence of the striping phenomenon could not be reached.

### Statistical analysis

The data was analyzed using the SPSS Sample Power Software (SPSS Inc., Chicago, IL, USA). One-way ANOVA and student’s t-test were utilized to evaluate the baseline demographic data and intraocular ocular pressure at each time point between the three groups. Kaplan-Meier survival analysis compared the cumulative probability of the qualified and complete success between the three groups. The chi-square test was adopted to calculate the qualified and complete success at postoperative 24 months and compare the percentage of filtering blebs with different morphology among the three groups. Two-way ANOVA was used for the multi-comparison of each time point. Univariate and multivariate linear regression analyses were utilized to investigate the correlation between bleb morphology parameters and IOP at 24 months postoperatively. A p-value < 0.05 was considered statistically significant. Intraclass correlation coefficients (ICC) were used to evaluate interobserver agreement on epithelial microcysts area, hyperreflective dots density, and connective tissue grade.

## Results

This study included 89 eyes from 85 patients. A total of 32 eyes were assigned to the Phaco-ExPRESS group; 25 eyes were assigned to the Phaco-Trab group, while another 32 eyes were assigned to the Trab group. The average follow-up periods were 26.5 ± 2.1 months, 27.5 ± 3.5 months, and 27.2 ± 3.0 months in the Phaco-ExPRESS, the Phaco-Trab, and the Trab group, respectively. Table 1 summarizes the demographics of the subjects in the three groups. Patients in the Phaco-ExPRESS and the Phaco-Trab group were older than the Trab group. All other baseline characteristics showed no statistical significance among the three groups. The change in IOP is presented in Table 2. The Trab group showed higher preoperative IOP than the other two groups (Phaco-ExPRESS vs. Trab, 21.15 ± 8.27 vs. 29.82 ± 11.32, *P* < 0.01; Phaco-Trab vs. Trab, 24.21 ± 7.80 vs. 29.82 ± 11.32, *P* = 0.01). However, no statistical significance was found in postoperative IOP between the three groups. For preoperative anti-glaucoma medications, the Phaco-ExPRESS and the Phaco-Trab group had fewer preoperative anti-glaucoma medications than the Trab group (Phaco-ExPRESS vs. Trab: 1.97 ± 1.12 vs. 3.00 ± 1.10, *P* < 0.01; Phaco-Trab vs. Trab: 2.24 ± 0.94 vs. 3.00 ± 1.10, *P* = 0.01). The Phaco-ExPRESS and the Trab group also showed fewer anti-glaucoma medications than the Phaco-Trab group at 24 months postoperatively (Phaco-ExPRESS vs. Phaco-Trab, 0.53 ± 0.84 vs. 1.32 ± 1.21, *P* = 0.04; Trab vs. Phaco-Trab, 0.72 ± 0.99 vs. 1.32 ± 1.21, *P* = 0.03).

**Table 1:** Demographic table

	Phaco-ExPRESS (n = 32)	Phaco-Trab (n = 25)	Trab (n = 32)	*P* value
Gender (M/F)	8/14	14/11	13/19	P = 0.96
Age (Mean ± SD)	74.19 ± 5.25	71.24 ± 8.94	57.21 ± 14.32	**†P < 0.01** †P = 0.39 **§P < 0.01**
MD (Mean ± SD)	-15...30 ± 9.00	-19.85 ± 9.67	-20.15 ± 8.37	†P = 0.27 §P = 0.23
PSD (Mean ± SD)	7.65 ± 3.53	7.27 ± 3.31	8.81 ± 4.30	†P = 0.99 §P = 0.90
BCVA LogMAR (Mean ± SD)	0.33 ± 0.23	0.45 ± 0.18	0.39 ± 0.24	†P::;0.11 §P = 071
CCT (Mean ± SD)	542.36 ± 21.81	540.20 ± 25.95	528.85 ± 31.11	†P = 0.99 §P = 0.24
*CID* Ratio (Mean ± SD)	0.84 ± 0.12	0.82 ± 0.13	0.87 ± 0.17	†P-0.99 §P = 098

The patient’s baseline characteristics. M/F: Male/Female; MD: mean deviation; PSD: pattern standard deviation, BCVA: best corrected visual acuity; CCT: central corneal thickness; C/D Ratio: cup to disc ratio, †: Phaco-Trab vs. Trab, ‡: Phaco-ExPRESS vs. Phaco-Trab, §: Phaco-ExPRESS vs. Trab. A p-value < 0.05 was considered as significant. Patients in the Phaco-ExPRESS and the Phaco-Trab group were older than the Trab group (Phaco-ExPRESS vs. Trab, *p* < 0.01; Phaco-Trab vs. Trab, *p* < 0.01). All other baseline characteristics showed no statistical significance among the three groups

**Table 2:** Pre- and Post-operative change of IOP and antiglaucoma medications in the three groups

	Phaco-ExPRESS (n = 32)	Phaco-Trab (n = 25)	Trab (n = 32)	*P* value
**PRE-OP IOP** **(Mean ± SD)**	21.15 ± 8.27	24.21 ± 7.80	29.82 ± 11.32	†P = 0.23 ‡**P = 0.03** **§P < 0.01**
**POST-OP 2 W IOP** (Mean ± SD)	9.57 ± 3.16	11.56 ± 3.56	10.02 ± 4.21	†P = 0.05 ‡P = 0.12 §P = 0.63
**POST-OP 1 M IOP** (Mean ± SD)	11.06 ± 3.15	12.81 ± 3.81	12.91 ± 4.47	†P = 0.10 ‡P = 0.93 §P = 0.06
**POST-OP 3 M IOP** (Mean ± SD)	12.51 ± 3.21	13.08 ± 4.05	13.10 + 4.58	†P = 0.59 ‡P = 0.99 §P = 0.56
**POST-OP 6 M IOP** (Mean ± SD)	12.49 ± 3.53	12.94 ± 3.54	12.54 ± 3.26	†P = 0.63 ‡P = 0.77 §P = 0.84
**POST-OP 12 M IOP** (Mean ± SD)	13.14 ± 3.69	13.62 ± 3.84	12.37 ± 3.53	†P = 0.63 ‡P = 0.25 §P = 0.47
**POST-OP 18 M IOP** (Mean ± SD)	12.71 ± 2.51	13.92 ± 3.87	12.93 ± 3.32	†P = 0.18 ‡P = 0.29 §P = 0.73
**POST-OP 24 M IOP** (Mean ± SD)	13.17 ± 3.48	18.00 ± 3.38	12.90 ± 3.08	†P = 0.85 ‡P = 0.91 §P = 0.74
**NO. of antiglaucoma medications at baseline** (Mean ± SD)	1.97 ± 1.12	2.24 + 0.94	3.00 ± 1.10	†P = 0.37 ‡P = 0.01 §P < 0.01
**NO. of antiglaucoma medications at** (Mean + SD)	0.53 ± 0.84	1.32 ± 1.21	0.72 ± 0.99	†P = 0.04 ‡P = 0.03 §P = 0.46

The IOP preoperatively and postoperative 2 weeks (POST-OP 2 W), postoperative 1 month (POST-OP 1 M), postoperative 3 months (POST-OP 3 M), postoperative 6 months (POST-OP 6 M), postoperative 12 months (POST-OP 12 M), postoperative 18 months (POST-OP 18 M), postoperative 24 months (POST-OP 24 M), number of preoperative and postoperative anti-glaucoma medications. †: Phaco-ExPRESS vs. Phaco-Trab, ‡: Phaco-Trab vs. Trab, §: Phaco-ExPRESS vs. Trab. A p-value < 0.05 was considered as statistically significant. For preoperative IOP, the Phaco-ExPRESS group showed lower IOP than the Trab group (*p* < 0.01). No statistical significance was found in postoperative IOP from 2 weeks to 24 months among the three groups. However, for preoperative anti-glaucoma medications, the Trab group showed a higher number of anti-glaucoma medications than the other two groups (*p* < 0.05). As for postoperative anti-glaucoma medications, no significant difference was found between the Phaco-ExPRSS group and the Trab group, whereas the Phaco-Trab group showed higher number of medications than the other two groups (*p* < 0.05)

### Control of IOP after surgery in three groups

**Fig. 2:**
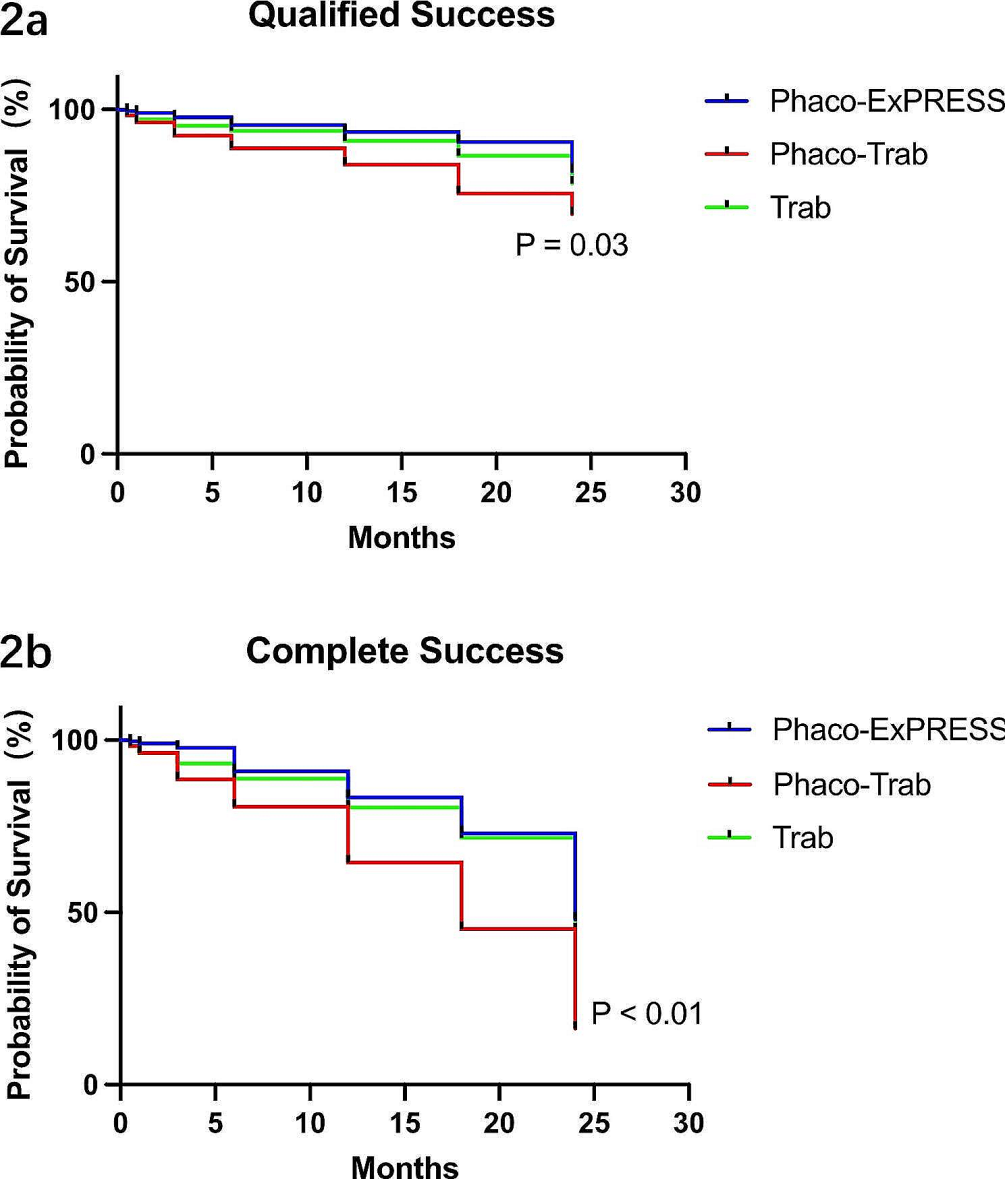
Kaplan-Meier survival analysis. The Kaplan-Meier survival analysis of qualified and complete success. Qualified success was defined as IOP < 18 mmHg with or without anti-glaucoma medication; complete success was defined as IOP < 18 mmHg without anti-glaucoma medication. *P* < 0.05 was considered as statistically significant. The results showed higher cumulative probability of qualified success in the Phaco-ExPRESS group than in the Phaco-Trab group. For complete success, both the Phaco-ExPRESS and the Trab group showed high cumulative probability of success than the Phaco-Trab group

Table 3 shows the success rate of the three groups at 24 months postoperatively. The qualified success rate showed no significant difference among the three groups at postoperative 24 months (*P* = 0.90). For complete success rate, the Phaco-Trab group showed a lower success rate than the other groups (Phaco-Trab vs. Phaco-ExPRESS, *P* = 0.01; Phaco-Trab vs. Trab, *P* = 0.04) at postoperative 24 months, while no significant difference was found between the Phaco-ExPRESS and the Trab group (*P* = 0.61). Figure 2 shows the result of the Kaplan-Meier survival analysis. The Phaco-ExPRESS group showed a higher cumulative probability of qualified success and complete success than the Phaco-Trab group, while the Trab group showed a higher cumulative probability of complete success than the Phaco-Trab group. When comparing the Phaco-ExPRESS group with the Trab group, no statistical difference was found in the cumulative probability of qualified or complete success at postoperative 24 months.

**Table 3:** Success rate at postoperative 24 months in three groups

	Phaco - ExPRESS (n = 32)	Phaco - Trab (n = 25)	Trab (n = 32)	*P* Value
Qualified success	90.6%	92.0%	93.8%	0.90
Complete success	65.1%	32.0%	59.4%	**0.03** + p = 0.01 ↕ p = 0.04 § P = 0.61

Compared the qualified success and complete success between the three groups at 18 months postoperatively. †: Phaco-ExPRESS vs. Phaco-Trab, ‡: Phaco-Trab vs. Trab, §: Phaco-ExPRESS vs. Trab. *P* < 0.05 was considered as statistically significant. No statistical significance was found in qualified success among the three groups. The Phaco-Trab group showed lower complete success rate than the other two groups at 24 months postoperatively (Phaco-Trab vs. Phaco-ExPRESS, *P* = 0.01; Phaco-Trab vs. Trab, *P* = 0.04)

### Assessment of filtering blebs

**Fig. 3:**
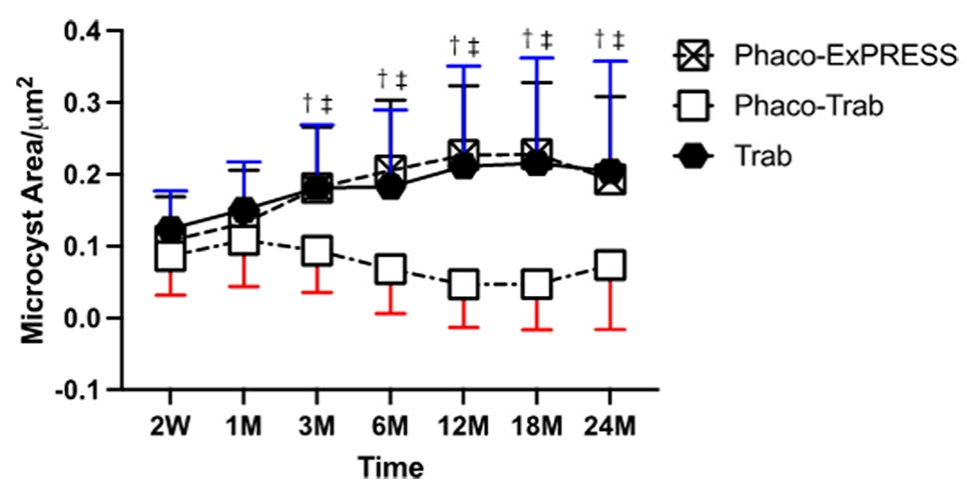
Epithelial microcysts area of filtering blebs. The epithelial microcysts area of the three groups at postoperative 2 weeks (2 W), 1 month (1 M), 3 months (3 M), 6 months (6 M), 12 months (12 M), 18 months (18 M), 24 months (24 M). *P* < 0.05 was considered as statistically significant. Statistically significant between Phaco-Trab vs. Trab group, Phaco-ExPRESS vs. Phaco-Trab group, and Phaco-ExPRESS vs. Trab group were marked as †, ‡, and §, respectively. The Phaco-ExPRESS group showed increased epithelial microcysts area from 1 month to 3 months and 6 months to 12 months (*p* < 0.05), and the Trab group showed increased epithelial microcysts area from 2 weeks to 3 months (*p* < 0.05) postoperatively and remained steady from 3 months to 24 months (*p* > 0.05). However, the Phaco-Trab group showed decreased epithelial microcysts area from 3 months to 12 months (*p* < 0.05). For comparison between the three groups, the Phaco-Trab group showed smaller microcysts area than the other two groups from postoperative 3 months to 24 months, while the epithelial microcysts area of the Phaco-ExPRESS group was similar to that of the Trab group from postoperative 2 weeks to 24 months

As shown in Fig. 3, in 24 months follow-up, the Phaco-ExPRESS, and the Trab group showed a statistically significant increase in mean epithelial microcysts area from 0.11 ± 0.06 µm^2^ to 0.19 ± 0.12 µm^2^ and from 0.13 ± 0.06 µm^2^ to 0.19 ± 0.15 µm^2^ in 1 µm^2^, from postoperative 2 weeks to postoperative 24 months respectively. However, the Phaco-Trab group decreased mean epithelial microcysts area from 0.09 ± 0.05 µm^2^ to 0.07 ± 0.09 µm^2^ in 1 µm^2^ from 2 weeks to 24 months postoperatively with no statistical significance. The Phaco-Trab groups showed significantly smaller mean epithelial microcysts area than the Trab and the Phaco-ExPRESS groups from 3 months to 24 months postoperatively. No significant difference in mean epithelial microcysts area was found between the Phaco-ExPRESS and the Trab group.

**Fig. 4:**
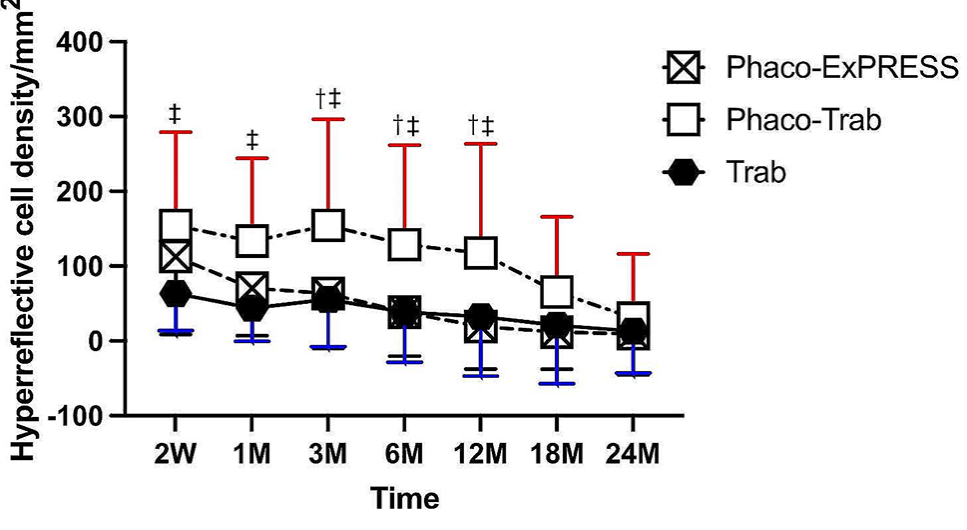
Density of hyperreflective dots in filtering blebs. The hyperreflective dot density of the three groups at postoperative 2 weeks (2 W), 1 month (1 M), 3 months (3 M), 6 months (6 M), 12 months (12 M), 18 months (18 M), 24 months (24 M). *P* < 0.05 was considered as statistically significant. Statistically significant between Phaco-ExPRESS vs. Phaco-Trab group, Phaco-Trab vs. Trab group, and Phaco-ExPRESS vs. Trab group were marked as †, ‡, and §, respectively. The Phaco-ExPRESS group showed decreased in hyperreflective dot count from postoperative 6 months to 12 months, while the Phaco-Trab group did not show decreased in hyperreflective dot until 12 months postoperatively. The Trab group showed no significant differences in hyperreflective dot count among different time points. For comparison between the three groups, the Phaco-Trab group showed higher hyperreflective dot count than the Trab group from 2 weeks to 12 months postoperatively. The Phaco-ExPRESS group showed less hyperreflective dot count than the Phaco-Trab group from 3 months to 12 months postoperatively

Figure 4 shows the density of hyperreflective dots in filtering blebs of the three groups. Even though both the Phaco-ExPRESS and the Phaco-Trab showed statistically significant decrease in hyperreflective dots from postoperative 2 weeks to 24 months, the hyperreflective dots of the Phaco-ExPRESS group were significantly decreased by 45.3% from postoperative 1 month to 6 months, while the Phaco-Trab group did not show a significant decrease in hyperreflective dots until 12 months postoperatively, of which the hyperreflective dots were decreased by 44.2%. When comparing the hyperreflective dots between the three groups, both the Phaco-Trab group and the Phaco-ExPRESS showed higher hyperreflective cell count than the Trab group at 2 weeks postoperatively (Phaco-Express vs. Trab: 112.70 ± 103.80 /mm^2^ vs. 63.48 ± 49.39 /mm^2^, *P* = 0.05; Phaco-Trab vs. Trab: 154.25 ± 125.14 /mm^2^ vs. 63.48 ± 49.39 /mm^2^, *P* < 0.01). Besides, the Phaco-Trab group also showed statistically higher hyperreflective dots than the Trab and the Phaco-ExPRESS group from 1 month to 12 months postoperatively.

**Fig. 5:**
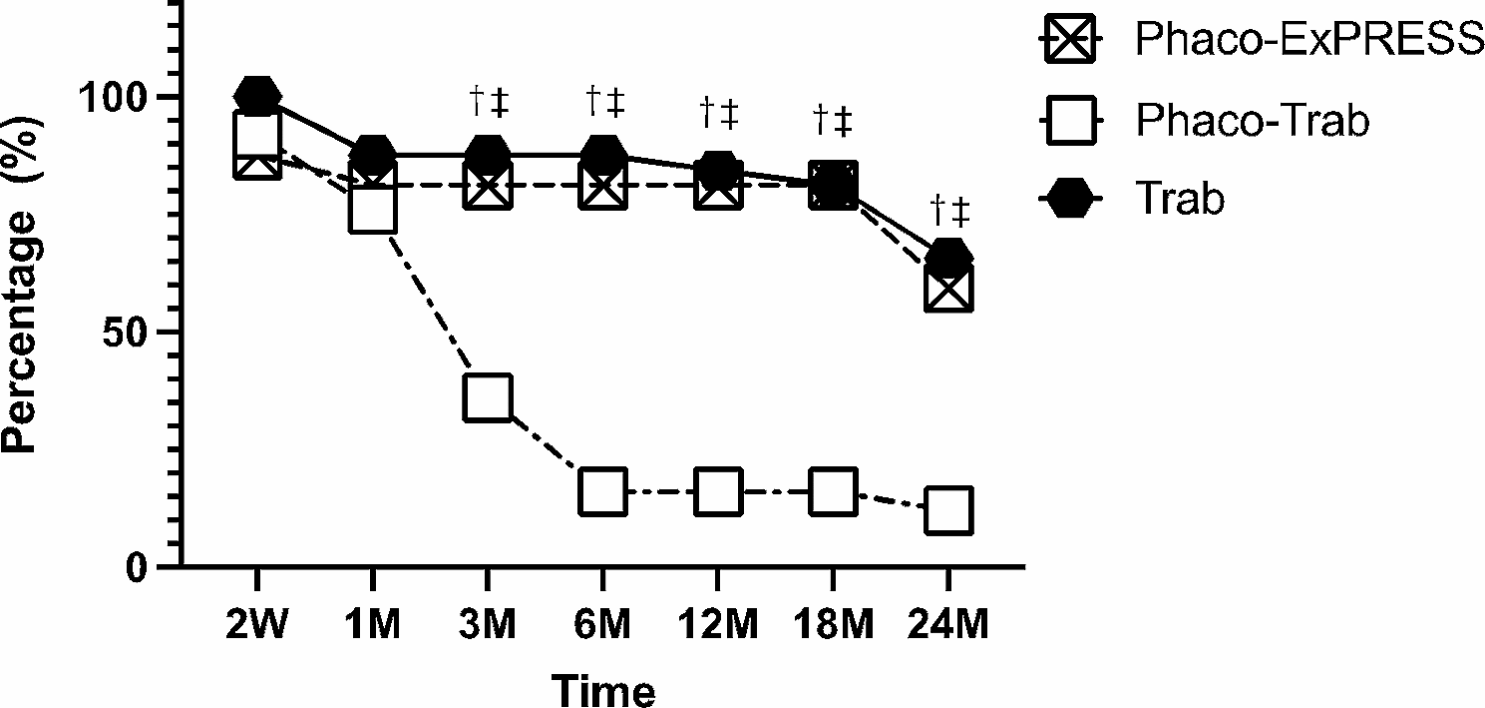
Percentage of filtering blebs with connective tissue grade ≤ 2. The percentage of filtering blebs with connective tissue grade ≤ 2 in the three groups at postoperative 2 weeks (2 W), 1 month (1 M), 3 months (3 M), 6 months (6 M), 12 months (12 M), 18 months (18 M), 24 months (24 M). *P* < 0.05 was considered as statistically significant. Statistically significant between Phaco-ExPRESS vs. Phaco-Trab group, Phaco-Trab vs. Trab group, and Phaco-ExPRESS vs. Trab group were marked as †, ‡, and §, respectively. The Phaco-ExPRESS and the Trab group showed a higher percentage of filtering blebs with connective tissue ≤ 2 than the Phaco-Trab group from 3 months to 24 months postoperatively

**Fig. 6:**
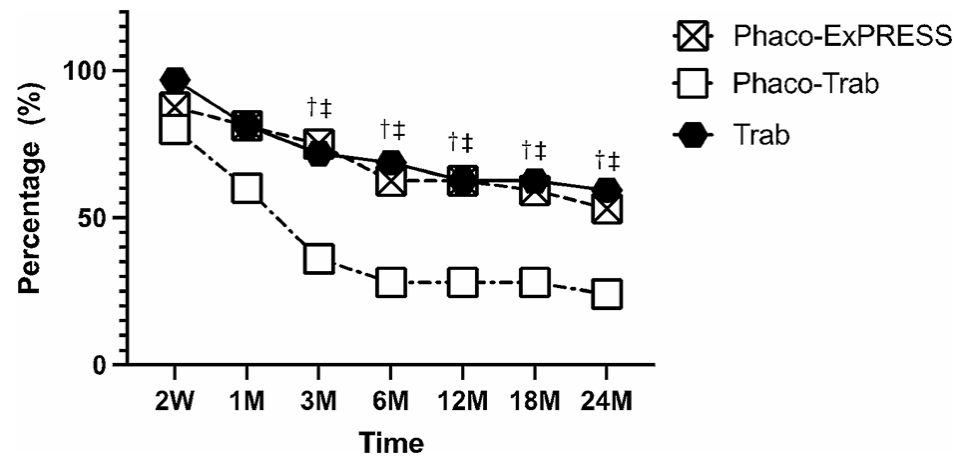
Percentage of filtering blebs with striping phenomenon. The percentage of filtering blebs with striping phenomenon in the three groups at postoperative 2 weeks (2 W), 1 month (1 M), 3 months (3 M), 6 months (6 M), 12 months (12 M), 18 months (18 M), 24 months (24 M). *P* < 0.05 was considered as statistically significant. Statistically significant between Phaco-ExPRESS vs. Phaco-Trab group, Phaco-Trab vs. Trab group, and Phaco-ExPRESS vs. Trab group were marked as †, ‡, and §, respectively. The Phaco-ExPRESS and the Trab group showed higher percentage of filtering blebs with striping phenomenon than the Phaco-Trab group from 3 months to 24 months postoperatively

Regarding the connective tissue density of the three groups, we calculated the percentage of filtering blebs with loose to mildly dense connective tissue networks (connective tissue grade ≤ 2). As shown in Fig. 5, the Phaco-ExPRESS and the Trab group showed 59.4% and 65.6% filtering blebs with connective tissue grade ≤ 2, respectively, while only 12.0% of filtering blebs in the Phaco-Trab met the criteria at 24 months postoperatively. From postoperative 3 months to 24 months, the Phaco-Trab group showed a lower percentage of filtering blebs with loose connective tissue than the other two groups. In accordance with the IVCM results, assessment by AS-OCT also showed a lower percentage of filtering blebs with the striping phenomenon in the Phaco-Trab group than the other groups from postoperative 3 months to 24 months (shown in Fig. 6). At postoperative 24 months, only 24.0% of filtering blebs in the Phaco-Trab group had striping phenomenon while 53.1% and 59.4% of filtering blebs in the Phaco-ExPRESS and the Trab group, respectively, had striping phenomenon. No significant difference was found between the Phaco-ExPRESS and the Trab group in connective tissue density and the presence of the striping phenomenon at each time point.

To better investigate the relationship between bleb morphology parameters and IOP outcome, we used age and anti-glaucoma medications at postoperative 24 months as covariates. We analyzed the correlation between IOP at postoperative 24 months and IVCM parameters and the presence of striping phenomenon (Table 4). We found that the absence of striping phenomenon at 12, 18, and 24 months postoperatively were associated with 1.848, 2.017 and 1.817 mmHg higher of postoperative IOP at postoperative 24 months. However, the IOP at postoperative 24 months did not show any correlation with the epithelial microcysts area, density of hyperreflective dots, or connective tissue grade at each time point.

**Table 4:** Multivariate linear regression according to IOP postoperative 24 months

	Coefficient	Standardized coefficient	95% CI	*P* value
**Epithelial microcysts area/µm**				
POST-2W	6.017	0.108	-6.688 to 18.723	0.35
POST-1M	3.194	0.068	-7.314 to 13.702	0.55
POST-3M	-1.716	-0.046	-10.239 to 6.808	0.69
POST-6M	-5.016	-0.165	-12.100 to 2.069	0.16
POST-12M	-4.225	-0.168	-10.123 to 1.673	0.16
POST-18M	-4.422	-0.181	-10.202 to 1.357	0.13
POST-24M	-5.271	-0.215	-11.164 to 0.621	0.08
**Density of hyperreflective dots/mm2**				
POST-2W	0.001	0.021	-0.006 to 0.008	0.85
POST-1M	0.003	0.063	-0.006 to 0.012	0.58
POST-3M	< 0.001	< 0.001	-0.007 to 0.007	0.99
POST-6M	< 0.001	-0.011	-0.008 to 0.008	0.93
POST-12M	< 0.001	-0.01	-0.008 to 0.007	0.94
POST-18M	-0.002	-0.048	-0.012 to 0.008	0.70
POST-24M	-0.008	-0.155	-0.019 to 0.004	0.18
**Connective tissue grade**				
POST-2W	-0.657	-0.135	-1.742 to 0.427	0.23
POST-1M	0.262	0.054	-0.844 to 1.368	0.64
POST-3M	-0.324	-0.087	-1.187 to 0.540	0.46
POST-6M	-0.248	-0.077	-1.013 to 0.516	0.52
POST-12M	-0.279	-0.090	-1.032 to 0.473	0.46
POST-18M	-0.124	-0.046	-0.779 to 0.531	0.71
POST-24M	0.080	0.025	-0.752 to 0.911	0.85
**Absence of striping phenomenon**				
POST-2W	-1.080	-0.105	-3.316 to 1.157	0.34
POST-1M	0.534	0.071	-1.138 to 2.206	0.53
POST-3M	1.168	0.173	-0.300 to 2.637	0.12
POST-6M	1.407	0.215	-0.017 to 2.832	0.05
POST-12M	1.848	0.283	0.450 to 3.246	**0.01**
POST-18M	2.017	0.309	0.629 to 3.406	**0.01**
POST-24M	1.817	0.278	0.380 to 3.254	**0.01**

Data are presented in coefficient (95% confidence interval). In the multivariate linear regression model, data is adjusted by age and postoperative anti-glaucoma medications number. A *p* < 0.05 are considered as significant. EM: epithelial microcysts area, HD: density of hyperreflective dots, CT: connective tissue grade, POST-2 W: postoperative 2 weeks, POST-1 M: postoperative 1 month, POST-3 M: postoperative 3 months, POST-6 M: postoperative 6 months, POST-12 M: postoperative 12 months, POST-18 M: postoperative 18 months, POST-24 M: postoperative 24 months

No serious choroidal detachment and corneal decompensation were found in the three groups. Conjunctival wound dehiscence was found in two patients (6.3%) in the Phaco-ExPRESS group, one patient (4.0%) in the Phaco-Trab group, and no patient in the Trab group. All of them underwent wound repair by suturing. Two patients (6.3%) in the Phaco-ExPRESS group and two patients (8.0%) in the Phaco-Trab group presented with shallow anterior chambers in the early postoperative period, and all recovered after conservative treatments. In addition, two patients (6.3%) in the Phaco-ExPRESS group, two patients (8.0%) patients in the Phaco-Trab group, and one patient (3.1%) in the Trab group underwent laser suture lysis. Postoperative needling with intra-bleb 5 fluorouracil was performed in two patients (6.3%) in the Phaco-ExPRESS and three (12.0%) in the Phaco-Trab. No additional glaucoma surgery was performed in the three groups.

To determine interobserver variability, two different observers combined and analyzed all three groups of patients. The ICC of epithelial microcysts area, hyperreflective dots, and connective tissue grade by the investigator (YQZ) and investigator 2 (CXL) were 0.725, 0.784, 0.864, and 0.953, respectively.

## Discussion

The management of coexisting cataracts and POAG is a common clinical challenge [2]. For the past twenty years, the development of cataract surgery has made it more and more popular for ophthalmologists to manage coexisting cataracts and glaucoma with one surgery. The meta-analysis showed that the IOP reduction in the combined surgery for cataract extraction and the trabeculectomy could result in 6–8 mmHg of IOP reduction in individuals, followed up for a mean of 1 to 2 years [35]. However, adding a cataract surgery to a trabeculectomy appears to diminish the IOP lowering effect of trabeculectomy alone by about 2 to 4 mmHg on average [35]. Lam et al. also showed that trabeculectomy alone could produce 50% of the complete success rate, while the Phaco-Trab reduced to only 19% at 5 years postoperatively [29]. The possible explanation is that the combination surgery intensifies postoperative inflammation and accelerates the fibrosis of filtering bleb, leading to unsatisfied IOP control [12, 29]. The Ex-PRESS glaucoma mini shunt implantation effectively reduced postoperative inflammation with a high success rate [18, 20–22]. Previous single-arm studies showed that the combined surgery of ExPRESS implantation and phacoemulsification had a qualified success rate (IOP ≤ 21 mmHg with or without antiglaucoma medications) of 76.9–83.5% at postoperative 3 years [36–39], while ExPRESS implantation alone could achieve a qualified success rate of 67–85% at postoperative 3 years [40, 41]. Two comparative studies compared the efficacy of ExPRESS implantation with or without phacoemulsification. Kanner et al. demonstrated that the implantation of ExPRESS alone and combined ExPRESS implantation with phacoemulsification showed comparable surgical success rate (94.8% vs. 95.6%) at 3 years after surgery [42]. Another study also showed that when combined with phacoemulsification, the Ex-PRESS mini shunt implantation can produce similar IOP reduction efficacy and success rate with the Ex-PRESS mini shunt implantation alone [30]. After 24 months of follow-up, we found that the Phaco-ExPRESS group had a similar cumulative probability of qualified and complete success with the Trab group, higher than that of the Phaco-Trab group. At 24 months postoperatively, the Phaco-ExPRESS group and the Trab group showed a lower number of anti-glaucoma medications than the Phaco-Trab group (Phaco-ExPRESS vs. Phaco-Trab, 0.53 ± 0.84 vs. 1.32 ± 1.21, *P* = 0.04; Trab vs. Phaco-Trab, 0.72 ± 0.99 vs. 1.32 ± 1.21, *P* = 0.03). Besides, the Phaco-ExPRESS group also showed a comparable complete success rate to the Trab group at postoperative 24 months (65.1% vs. 59.4%, *P* = 0.61). Therefore, the combination of the phacoemulsification with the ExPRESS mini shunt implantation had similar reducing IOP efficacy to the trabeculectomy alone when compared with the combination with the trabeculectomy, could provide better IOP control and less postoperative anti-glaucoma medications.

To better illustrate the differences between the Phaco-ExPRESS, the Phaco-Trab, and the Trab, we observed microstructural features of bleb morphologies of three surgical methods by IVCM. The epithelial microcysts area, the density of hyperreflective dots, and connective tissue density were evaluated quantitatively. Epithelial microcysts are thought to be the potential channels for the passage of aqueous humor after the formation of filtering blebs. They are presumed to be a positive predictive factor for functioning blebs [43, 44]. In our study, the quantitative analysis of microcysts area demonstrated an increase in microcysts area from postoperative 2 weeks to 24 months in 71.9% and 62.5% of filtering blebs in the Phaco-ExPRESS and the Trab group, respectively. No significant difference between these two groups was found in the epithelial microcysts area. In contrast, 76.0% of filtering blebs in the Phaco-Trab group showed a decrease in the micro cysts area from postoperative 2 weeks to 24 months. The Phaco-Trab group also showed a smaller microcyst from 3 months to 24 months postoperatively than the Phaco-ExPRESS and Trab groups. In general, with a larger microcyst area, which was similar to filtering blebs of the Trab group, filtering blebs of the Phaco-ExPRESS could be more potent than that of the Phaco-Trab group.

Persistent inflammation with deposition of fibrous tissue in the subconjunctiva is the most crucial cause that leads to fibrosis and failure of filtering blebs [45]. In our previous study, the hyperreflective dots presented in IVCM had been identified as neutrophil- and monocyte-like cells by Giemsa staining [27]. In this study, 87.5%, 84.0%, and 93.8% of filtering blebs in the Phaco-ExPRESS, the Phaco-Trab, and the Trab groups showed a decrease in density of hyperreflective dots from postoperative 2 weeks to 24 months. However, for the Phaco-ExPRESS and the Trab group, multiple comparison analysis showed that the density of hyperreflective dots started to decline as early as 6 months postoperatively, while the Phaco-Trab group did not show a decrease in density of hyperreflective dots until 12 months postoperatively, which may precipitate persistent inflammation of the filtering blebs. Besides, a higher density of hyperreflective dots was found in the Phaco-Trab group than the other groups except for 18 months and 24 months postoperatively, while hyperreflective dots were similar between the Phaco-ExPRESS and the Trab group. Our results are in accordance with a previous study, which indicated that the Ex-PRESS glaucoma mini shunt implantation was a surgical procedure that can induce less inflammation and vascularity of filtering blebs [16, 17]. The presence of inflammatory cells in the subconjunctival can lead to apoptosis of goblet cells around the microcysts and modification of fibrous tissue, resulting in smaller microcysts area and fibrosis of filtering blebs [46].

The blebs with loose to mildly dense connective tissue on IVCM have better-filtering function [34]. Showing a similar percentage of filtering blebs with the striping phenomenon and loose to mildly dense connective tissue, the blebs morphology of the Phaco-ExPRESS was similar to the Trab group in connective tissue density. The striping phenomenon obtained by AS-OCT was considered a positive marker to predict good IOP control post-trabeculectomy [34]. Narita et al. found that the presence of striping phenomenon at postoperative 2 weeks can predict reasonable control of IOP at 1-year post-trabeculectomy [34]. Our previous study also found the striping phenomenon as a characteristic of functioning blebs [27]. Consistent with this, we also found that the absence of striping phenomenon at postoperative 12, 18, and 24 months is associated with 1.85 mmHg, 2.02 mmHg, and 1.82 mmHg of increase in IOP, respectively, at postoperative 24 months. Besides, we also found that a higher percentage of filtering blebs with striping phenomenon was found in the Phaco-ExPRESS and the Trab group than in the Phaco-Trab group. In contrast, the percentage of filtering blebs showing striping phenomenon in the Phaco-ExPRESS group was comparable to the Trab group. These results strongly support that the filtering blebs of the Phaco-ExPRESS rather than the Phaco-Trab were more likely to form functioning blebs with sufficient IOP control.

The major limitation of our study is its retrospective design. A larger-scale prospective study is required to further verify the current findings in Chinese and other populations. Besides, the patients of the Trab group were younger. They had higher preoperative IOP than the other two groups, which may affect the outcome of the comparison between the three groups. Notably, filtering blebs of younger patients had more severe postoperative inflammation and a greater tendency to fibrosis, which may compromise the long-term effect of the efficacy of IOP control. Besides, some patients may undergo additional anti-glaucoma medications to reach the target IOP, including prostaglandin, which can increase the inflammation at the conjunctiva. The increase in inflammation may also affect the structure and function of filtering blebs.

In conclusion, the Phaco-ExPRESS and the Phaco-Trab are equally effective in managing POAG in 24-month follow-ups. However, the filtering blebs generated by the Phaco-ExPRESS were similar to that of the trabeculectomy, with wider bleb morphology, larger microcyst area, lower density of inflammatory cells, and looser connective tissue than that of the Phaco-Trab. It indicated that the Phaco-ExPRESS could be more potent than the Phaco-Trab when managing patients with coincident POAG and cataract.

## Data Availability

All data generated or analyzed during this study are included in this article. Further inquiries can be directed to the corresponding author.
